# Contemporary Knowledge Workers and the Boundaryless Work–Life Interface: Implications for the Human Resource Management of the Knowledge Workforce

**DOI:** 10.3389/fpsyg.2018.02414

**Published:** 2018-11-30

**Authors:** Justin Craig Field, Xi Wen Chan

**Affiliations:** ^1^UNE Business School, University of New England, Armidale, NSW, Australia; ^2^School of Management, College of Business, RMIT University, Melbourne, VIC, Australia

**Keywords:** work–life theories, boundaryless work–life interface, knowledge workers, human resource management, organizational psychology

## Abstract

In the last decade, knowledge workers have seen tremendous change in ways of working and living, driven by proliferating mobile communication technologies, the rise of dual-income couples, shifting expectations of ideal motherhood and involved fatherhood, and the rise of flexible working arrangements. Drawing on 54 interviews with Australian knowledge workers in the information technology sector, we argue that the interface between work and life is now blurred and boundaryless for knowledge workers. By this, we mean that knowledge workers are empowered and enslaved by mobile devices that bring work into the home, and family into the workplace. Knowledge workers take advantage of flexible working to craft unique, personal arrangements to suit their work, family, personal and community pursuits. They choose where and when to work, often interweaving the work domain and the home–family domain multiple times per day. Teleworkers, for example, attain rapid boundary transitions rending the work–home boundary, thus making their experience of the work–life interface boundaryless.

## Introduction

Ways of working and living have changed dramatically in post-industrial economies in the last decade. First, proliferating information and communication technologies (ICT), often mobile, are connecting people, but also intensifying work beyond traditional offices and working hours (Ciolfi and Lockley, 2018). Second, more women are working. Dual-income couples are now the norm (Abele and Volmer, 2011). Third, expectations of parenthood are changing. Mothers are working more, while balancing parenting with working. Fathers are increasingly more involved in parenting, shifting from exclusively being breadwinners (McGill, 2014). Finally, companies are offering flexible working arrangements (FWAs), so that employees can vary the time, schedule and location of work, to suit their needs.

Knowledge work is dominating in post-industrial economies. Knowledge work involves manipulating and transmitting ideas, rather than goods. In particular, knowledge workers use ICT to exchange meaning. Knowledge processes are unconstrained, occurring in any location and at any time (Nelson et al., 2017), but they are subject to the influence of organizational culture, technological support, and supervisory arrangements, as well as the agency of knowledge workers to subvert or comply with organizational norms.

Considering rapid technological change, demographic change, societal change and the rise of knowledge work, we propose a new appreciation of the boundaryless work–life interface for flexible knowledge workers. Despite intense work and non-work activities, we contend flexible knowledge workers do not emphasize the distinction between work and non-work. Rather, they work at any place and time, but also manage to achieve harmonious balance in their lives. Existing work–life theories do not present the work–life experiences of flexible knowledge workers accurately. Hence, we describe a richer conceptualisation of the work–life interface for this group and discuss implications for human resources (HR) policies and management methods in the digital economy.

## Existing Theories on the Work–Life Interface

Work–life theories can be classified into three streams: (1) negative side of the work–life interface, stemming from role strain theory, (2) positive side of work–life interface, stemming from role accumulation theory, and (3) blurred boundaries between work and non-work, stemming from boundary and border theory.

### Negative Side of the Work–Life Interface

Role strain theory, which originated from Goode’s (1960) scarcity perspective, contends that multiple roles lead to role strain and subsequently interrole conflict (work–family conflict) as it becomes difficult to perform each role due to conflicting demands on time, energy and attention among the roles (Greenhaus and Beutell, 1985). Defined as “an individual’s experience that work and family roles are incompatible in some respect, as a result of which participation in one role is made more difficult by virtue of participation in the other role” (Greenhaus and Beutell, 1985, p. 77), work–family conflict had dominated work–life research owing to the rise of dual-income households with children. However, Marks (1977) argued that role strain was not a result of incompatible role demands, but by role imbalance, as there is a difference in the importance of roles assumed. Marks (1977) added that no role strain would occur if all commitments were equally positive or negative. Barnett and Hyde (2001) also reasoned that having several roles was not the issue, it was the quality and combination of roles that contributed to role strain.

### Positive Side of the Work–Life Interface

As research on work–family conflict matured, Greenhaus and Powell (2006) responded to calls to examine the positive side of the work–life interface. Drawing on role accumulation theory, Greenhaus and Powell (2006, p. 73) proposed the concept of work–family enrichment, defined as “the extent to which an individual’s experiences in one role improve his or her quality of life in other roles.” Role accumulation theory was jointly developed by Sieber (1974) and Marks (1977), both of whom argued that having multiple roles is more rewarding than stressful, and that the more roles individuals took on, the more resources they possessed, and the more opportunities they were exposed to. Research on work–family enrichment increased drastically in the past decade in large part due to the positive psychology movement and demographic trends which have given rise to workplace policies that seek to enhance employees’ work and life (Brough and O’Driscoll, 2015). The strongest criticism on work–family enrichment is it does not acknowledge the negative side of the work–life interface, which many researchers argue cannot be dismissed since people tend to struggle with managing their work and non-work responsibilities.

### Blurring of Boundaries Between Work and Life

In this research, we use “work–life” as opposed to “work–family” or “work–home” to acknowledge non-work roles (e.g., community, social and personal pursuits) (Moen, 2011). Alongside work–family enrichment and conflict are several theories on work–life balance, including Ashforth et al.’s (2000) boundary theory and Clark’s (2000) border theory. However, research on work–life balance has not advanced theoretically because of inconsistent definitions of work–life balance (Kalliath and Brough, 2008). Border theory and boundary theory contribute to the study of work–life linkages by describing how varying levels of work–life integration affect well-being, and addressing how people construct and cross boundaries between work and life.

#### Boundary Theory

Boundary theory focuses on the meanings people assign to work and life (Nippert-Eng, 1996) and the ease and frequency of transitioning between roles (Ashforth et al., 2000). Boundaries are clearer when roles are separated, while role transitions are easier when roles are integrated. Role blurring is the experience of confusion or difficulty in distinguishing work from non-work roles, especially when roles are highly integrated (Desrochers et al., 2005). Boundaries can be classified by their flexibility and permeability. Flexibility is the “extent to which the physical time and location markers, such as working hours and workplace, may be changed”; permeability is “the degree to which a person physically located in one domain may be psychologically concerned with the other” (Hall and Richter, 1988, p. 215). Roles can be arranged along a segmentation–integration continuum: when role boundaries are inflexible and impermeable, the roles are segmented; when boundaries are flexible and permeable, roles are integrated (Ashforth et al., 2000). Studies (e.g., Hyland and Prottas, 2017) using boundary theory have found that permeability is asymmetrical—work demands tend to spill over into non-work domains. Individuals with strong segmentation preferences face challenges crossing boundaries; while those with strong integration preferences face challenges in creating and maintaining boundaries. Drawing on Weiss and Cropanzano (1996) affective events theory, Hunter et al. (2017) extended boundary theory by incorporating goal obstruction as the explanatory mechanism linking work-to-family conflict and job satisfaction. Specifically, boundary violation events (e.g., taking a phone call from a family member at work) occurring at work were appraised as obstructing one’s work goals due to time and attention redirected from meeting work goals to meeting family needs.

##### Boundaryless work–life interface of knowledge workers

Knowledge work tends to involve a high degree of both flexibility and permeability, facilitating role blurring or, increasingly, a state of “boundarylessness” (Albertsen et al., 2010). Unlike traditional “9-to-5” jobs, knowledge work is characterized by a less rule-based and more flexible regulation, especially in terms of time, space, and jobscope (Allvin, 2008). That is, knowledge workers typically have individualized schedules, temporal and geographical flexibility, and more job autonomy (Albertsen et al., 2010). “Boundarylessness” does not necessarily suggest the complete absence of boundaries between different life domains, but it illustrates weak to virtually absent domain boundaries (Ezzedeen and Zikic, 2017). Since the concept of “boundarylessness” is a new and novel phenomenon, we focus on understanding the boundaryless nature of knowledge workers’ work–life interface. Accordingly, the pursuit of empirical observations is important as it contributes to theory development and decision-making for employees, managers and organizations (Albertsen et al., 2010).

#### Border Theory

Border theory is devoted only to work and family domains. Work–family balance is the outcome of interest, defined as “satisfaction and good functioning at work and at home, with a minimum of role conflict” (Clark, 2000, p. 751). It differs from boundary theory in that definition of borders includes psychological categories and also tangible boundaries that divide the time, place and people associated with work and life. Borders are characterized by their strength, from weak to strong. Weak borders are more likely to be permeable and flexible, facilitating blending between roles. Strong borders are more likely to be inflexible and impermeable, preventing role blending (Clark, 2000). Border-keepers are members of a domain who are influential in defining the border and the domain. Conflict may arise when border-keepers and border-crossers do not agree on the exact boundaries of a domain. They may also disagree about the flexibility and permeability of the boundary.

### Research Question

In this study, our goal is to illuminate the poorly understood boundaryless nature of knowledge workers’ work–life interface. Existing work–life theories do not adequately and accurately account for knowledge workers’ work and non-work experiences. Therefore, we seek a deeper understanding of how flexible knowledge workers perceive and navigate their life domains, and to discover various contextual and socio-cognitive factors that influence their perceptions and decisions. Therefore, a qualitative design is particularly apposite for our research. We interpretively explored three questions: (1) How do flexible knowledge workers perceive, think about, and experience the interface between their work and non-work domains? (2) How do flexible knowledge workers perceive and manage role boundaries between their work and non-work domains? (3) What contextual and socio-cognitive factors account for flexible knowledge workers’ differing perceptions and decisions when managing their work–life interface? In sum, we qualitatively explored the perceptions and decisions of flexible knowledge workers with the understanding that these have implications for their individual, work and family functioning (Ashforth et al., 2000).

## Methods

To answer our research questions, we conducted an exploratory case study, using an embedded single-case design (Yin, 2009) to examine knowledge workers’ perceptions about balancing work and life. Each participant represented an embedded sub-case within the case study. We adopted an interpretive research approach, giving voice to participants’ interpretations and perceptions of the work–life interface. The participants’ point of view is the foundation of our analysis. This section provides a detailed description of our method, to support dependability and transferability (Lincoln and Guba, 1985).

### Data Collection

The research site was the Australian affiliate of a multi-national technology company (referred to as “Tech”). Tech had around 130,000 employees worldwide at the time of the study, with around 2,100 employees in Australia. Access to employees at the research site was negotiated with the HR director. Before we approached Tech, we sought ethics approval from the University of New England’s Human Research Ethics Committee. The HR director allowed us to gather data in two ways. First, semi-structured interviews were the principal source of information about participants’ thoughts, feelings and perceptions about work–life balance and how they arranged their working and personal lives. Confidentiality was protected by a written informed consent agreement with each participant. Second, we gathered policy documents, people directory entries and corporate broadcast emails from Tech’s intranet, and we downloaded Tech’s statutory reports to government agencies, as triangulation sources. In addition, the principal researcher wrote field notes after each interview to document researcher responses and states, using ongoing reflective commentary (Shenton, 2004) to capture assumptions, emotional states and possible bias.

### Semi-Structured Interviews

The lead researcher conducted all interviews to maintain a consistent data collection approach. He conducted pilot interviews with five participants drawn from Sales, Human Resources and Research & Development departments at Tech. Pilot participants used FWAs in different ways: three were teleworkers, one was part-time, and one was full-time, about to begin parental leave. The purpose of the pilot interviews was to test the interview template (see Appendix 1) against the research questions, across different jobs and different demographic attributes, and to enhance dependability by following a consistent procedure, per guidelines of Miles et al. (2014).

We developed the interview template for the pilot interviews from a study of the literature, focusing on satisfying the research questions. Prior to the pilot interviews, we reviewed the interview template with academic colleagues, to enhance objectivity and confirmability (Lincoln and Guba, 1985). Sample questions included: What triggered your request for flexible working? How do you balance work versus home and family? How do you define what is work time and what is non-work time?

Following the pilot interviews, the interview template was revised with supplementary questions. For teleworkers, we found it useful to ask: Do you ever work very early or very late? Is this your choice or has your manager directed you? How do you blend work tasks and home or childcare tasks, when working from home? For part-time participants, we asked: To what extent do you work on a non-working day? Is this your choice or has your manager directed you? We also found it useful to ask all participants about availability: When do you make yourself strictly unavailable? Do you ever disconnect? These questions explored richer detail about participants’ methods of forming and dismantling boundaries, and their perceptions of organizational norms and their personal responses to such expectations.

### Document Gathering

We gathered relevant documents from Tech’s intranet, including people directory entries for all participants, copies of published policies, and corporate broadcast emails from business leaders or Tech HR team. We also gathered Tech’s statutory reports to the Workplace Gender Equality Agency.

### Sampling and Saturation

To identify participants accessing FWAs, Tech’s HR team provided reports listing Tech employees with part-time hours, with flexible or remote working arrangements, and with completed leaves of absence (other than annual and sick leave). We used non-probability purposive and snowball sampling techniques (Bernard and Ryan, 2010). We used the reports to identify the population of all employees using FWAs, and we invited all employees in the population to participate in interviews. We also asked Tech HR managers and interviewees to recommend other employees who might have relevant experiences to share. This was productive because we discovered some employees using informal FWAs (e.g., working at home as a personal arrangement with the manager) did not appear on Tech’s official records.

Overall, we interviewed 54 participants at Tech (Field, 2017). Participants fell into three categories: part-time, teleworkers, and sabbaticals. Some participants had multiple experiences, so they fell into multiple categories. Participants represented 39% of all part-time employees at Tech Australia, and 40% of all teleworkers at Tech Australia, giving credibility and depth to the findings of this case study. Because sabbaticals were infrequent and episodic, it was not possible to calculate a participation rate.

Saturation for part-time participants was achieved after 12 of 24 interviews. After the twelfth interview, we heard repeated themes of work expanding into non-work time and efforts to juggle work and family. Saturation for teleworker participants was achieved after 15 of 30 interviews. After the fifteenth interview, we heard repeated themes of blending work and home/family during the work day and being available outside of conventional working hours. Turning to sabbatical participants, it was not possible to establish whether saturation was reached. Only five interviews were conducted, because employees going on sabbaticals are rare at Tech.

### Data Preparation

All recorded interviews were transcribed into text files (one participant did not consent to audio recording). We provided the text files to participants for verification, if they had made this request before interview, to enhance credibility and authenticity of the study (Miles et al., 2014). During transcription, the lead researcher recorded transcription memos to capture generative insights, connections and themes (Sunstein and Chiseri-Strater, 2012). Each transcript was made anonymous by introducing pseudonyms and code numbers for each participant. We used MAXQDA for data coding and analysis, and uploaded interview transcripts, field memos, transcription memos, and gathered documents. The data corpus consisted of 53 interview transcripts and one interview summary (Field, 2017), plus 177 other documents including field notes, transcription memos, coding memos, company policies, company statutory reports, company emails and people directory entries. There was a total of 473,206 words in interview documents, and 106,466 words in the other documents.

### Data Analysis

We use the technique of thematic qualitative text analysis (Kuckartz, 2014) to examine common elements between participants and groups, differences between participants and groups, and relationships within the data. The principal unit of analysis was each participant’s interview transcript. We read through each transcript closely multiple times, identifying segments addressing our research questions.

We began data analysis with a primarily deductive approach, in order to examine participant perceptions in light of role strain theory, role accumulation theory, boundary theory and border theory. We developed thematic categories (Kuckartz, 2014), displayed in Table 1, then coded all interviews using the broad thematic categories. We chose the unit of coding to be at the paragraph level, in interview transcripts. We also wrote individual case summaries.

**Table 1:** Deductive coding and analysis from literature review and theory.

Theoretical area	Category	Codes
Role strain theory	Work–life conflict	• Work conflict at home/family
		• Home/family conflict at work
Role accumulation theory	Work–life enrichment	• Work enrichment at home/family
		• Home/family enrichment at work
Boundary theory and Border theory	Boundaries and borders	• Defining work domain boundaries
		• Defining home/family boundaries
		• Crossing boundaries
		• Perceptions of boundary keepers
		• Perceptions of boundary crossers

In the second cycle of coding, we retrieved all segments within a category, then used an inductive method, in alignment with our research questions, to formulate sub-categories from the data. For example, when investigating ‘Crossing boundaries’ and ‘Work conflict at home/family’ for part-time participants, we coded for the health status of dependent children, the nature of the day (working or non-working), the clock time (within or beyond conventional working hours), and the organizational hierarchical status (e.g., vice president or individual contributor) of the co-workers making contact.

To enhance credibility, preliminary themes and findings were shared with academic colleagues and discussed at length, to challenge the analysis, using the peer debriefing method (Kuckartz, 2014). The purpose of this debate was so that findings would not rely on the interpretations of a single analyst alone, enhancing trustworthiness of the analysis (Miles et al., 2014). We also used negative and deviant case analysis (Richards and Hemphill, 2018), aiming to find exception cases in the data set, to bolster our understanding of participant perceptions of their work–life interface. We conducted extensive cross-case analysis, comparing cohorts (part-time, teleworkers, sabbaticals), gender, age groups, career level (individual contributors, front-line managers, middle managers), participant tenure with manager, and participant tenure with Tech.

### Conducting Research as a Privileged Insider

During data collection and analysis, the lead researcher was an employee of Tech. Insider status afforded privileged access to the research site and to gatekeepers within the organization. Insider status also provided an extensive network of acquaintances and co-workers as key participants of the case study. Furthermore, tacit and intrinsic knowledge of Tech’s organizational culture was useful to establish shared cultural membership with participants. Using this knowledge, the lead researcher established trust and rapport with participants, enhancing each participant’s sense of freedom to voice their stories. Conversely, the researcher’s insider status raised fears and risks for some participants. Specifically, some participants were concerned that their stories, employment history or family arrangements were unique, which allowed others to identify them. Some feared their reputation might be damaged or feared their private opinions about co-workers might damage relationships. We addressed these fears by emphasizing arrangements for confidential treatment of interviews, anonymity and provision of pseudonyms.

## Findings

### Sample Demographic Characteristics

The sample consisted of 54 flexible knowledge workers. The sample was 62.96% female and 37.04% male. Mean age of participants was 43.24 years (*SD* = 7.49 years). Mean organizational tenure was 10.12 years (*SD* = 6.55 years). The number of individual contributors was 41; the number of front-line managers was seven and the number of middle managers was six. Five participants were single, six participants had a partner and no dependents, and 43 participants had a partner and dependent children. Dual-earner couples made up 75.93% of the sample.

### The Case for the Boundaryless Work–Life Interface

From our analysis, we argue that flexible knowledge workers perceive the work–life interface as fuzzy and boundaryless. Work–life balance is different for every person: subjective perceptions of demands originating from work, home–family and other life domains define how individuals measure the success of FWAs.

Despite intense work and non-work activities, we found that participants barely distinguished between work and non-work. Our finding is in line with the research findings of Hill et al. (2003) who found that virtual office workers tend to have difficulty knowing when they are at work and when they are home, due to the lack of externally imposed physical boundaries. Facilitated by ICT, our participants worked in many different locations at various times. They leveraged FWAs to take care of home–family concerns during office hours. Our participants also overwhelmingly favored an integration preference, with highly flexible and permeable boundaries. *Ronald* showed how he permitted family concerns to cross into the work domain, leveraging spatio-temporal flexibility.

Ronald: I’m present [for work] if I need to, if I get a phone call from school and my wife is at work, and one of the kids needs to be picked up because they’re not feeling well, it has allowed me that flexibility … for me to go and pick them up. *(Individual contributor, full-time, age 35–39, teleworker some of the week)*.

Whereas segmentation was the norm decades ago (Nippert-Eng, 1996), now integration is the norm for flexible knowledge workers. Participant expectations about where and when to work are boundaryless—and the physical and technological barriers that surround these expectations have been abolished. Boundaries are so permeable they do not even matter, reinforcing fuzzy boundarylessness. With ICT in hand, knowledge workers move seamlessly from work demands to home demands and back again, not paying much attention to boundaries. *Katrina* focused on her children during part of the day. When she missed a work telephone call, she quickly returned it, demonstrating high permeability and high integration.

Katrina: I’ll answer it, if I really can’t answer it I will let it go to voicemail and then I’ll call them back, 10 min later when, when I can. But generally speaking I will just, a child in one hand and I’m on the phone to them. *(Individual contributor, part-time, age 25-29, office based).*

When working at home, some or all of the week, or participating in global projects collaborating across time zones, participants found they needed to work earlier or later than core working hours. Yet, during the day, they used time for home–family tasks. By doing this, participants enacted home devotion and simultaneously work devotion (as defined by Blair-Loy, 2003). They were involved parents, yet also appearing as ideal workers to their managers and co-workers. They could also work the same or more number of hours each day, intersperse several hours of quality family time, without impacting their work–life balance (Hill et al., 2003). *Anastasia* illustrated how she interwove between work and family during core working hours, but then extended the working day into the evening.

Anastasia: Ok, well, the girls have got something on at assembly today. I’m just going to go. So I block out my diary and I go to the assembly and enjoy it and I am present for them and then I come home and log back on. Now I might have to work a few extra hours that night after they go to bed. You do it. You just juggle it. Some days I don’t even work my core hours. *(Individual contributor, age 35–39, teleworker some of the week).*

Likewise, *Thomas* took an active role as an involved father, in the late afternoon, but worked intensely during early mornings and late at night.

Thomas: One of the things that I’ve always been very careful about is to not let the work–life balance get out of kilter. Given that I’ve got two young kids, what works for me exceptionally well is the fact that because I’ve got a global job where I’m on the phone from, most days, from 06:00 till 08:00 or 09:00 in the morning, and then I’m on the phone again from 20:00 at night through till 22:00 or 23:00, means that I essentially don’t do any work in the early afternoons, which is 15:00 in the afternoon through until about 20:00 at night, so when the kids get home from school. *(Individual contributor, full-time, age 45–49, teleworker all of the week).*

We argue there is reduced work–life conflict in this state of boundarylessness. *Thomas* specifically mentioned leveraging FWAs to manage his work–life balance, to fit his expectations. Because flexible knowledge workers have more freedom to choose working arrangements to suit home–family arrangements, we argue they use the technique of crafting their work–life interface to suit their requirements. We found work–life conflict was present in a small way, but not regarded as excessive by participants. The autonomy afforded by FWAs gave participants control over work *and* home–family, thus they were able to meet demands from all domains under conditions of apparent work–life balance.

Participants did not take a pure work-oriented view of the world. They did not simply regard themselves as employees, above all else, as *Anastasia* and *Thomas* demonstrate. We contend that individuals make work–life balance decisions across their life-space, including all their roles (e.g., students, workers, spouses, homemakers, parents, and citizens). Their decisions vary over the life course, as they move across generations and work and home demands shift in priority. Events such as marriage, divorce, childbirth, promotion or relocation shift perceptions about home–family demands and work demands. Choices about whether to use FWAs are not solely governed by perceptions at work, and penalties and benefits from flex-work, but are also governed by perceptions at home, and associated penalties and benefits.

Organizational expectations played a significant role in shaping participant perceptions about boundaries. We observed that managers hardly ever requested extended work hours from participants. Instead, individuals worked where and when they perceived it was necessary, either to complete their workload (especially so for managers), or, to collaborate with co-workers in distant time zones. *Ethan* explained how he perceived Tech’s organizational culture and the demands of his intense workload as a middle manager.

Ethan: And the company doesn’t set it as an expectation. They don’t expect me to sit, or the hundreds of other people that sit on their sofas at night, doing email for the day. It’s not expected, right. But it’s not discouraged. […] It’s a self-driven, there’s no expectation, other than your personal drive to be on top of or in front of or caught up on, or, but it’s, you’re never in front of, right. You’re always […] You’re never in front, yeah. So it’s just about keeping head above water in some respects, right.

*Lauren* explained her views about the mistaken freedom of working from home, and the requirement to be constantly available, enforced by her manager’s behavior. Organizational norms were powerful in shaping acceptable and unacceptable behaviors for participants.

Lauren: No, because if you do that, there will be him [Lauren’s manager] asking, where are you? You should always be available. So there was no hiding. There was absolutely no hiding. So even though that people say that you work from home and you’ve got all this freedom, it’s nothing like that. On the contrary, in fact. Because you felt like you had to be constantly by your computer so you can answer your chat right away, or your phone right away. Because if there was a delay there’s always that worry at the back of your head, oh, do they think I’m not working? *(Individual contributor, full-time, age 40–44, teleworker some of the week).*

So, fuzzy boundarylessness has a dark side. Participants framed use of ICT to craft reputations of high availability as a personal choice. Yet, participants were responding to strongly held behavioral norms in organizational culture. There was little resistance to working outside conventional hours—it seemed so ‘normal’. But, per *Lauren*’s remarks, participants were not truly free to decide working hours independently. Constant availability for work has become a proxy for organizational commitment. Virtual displays of employee engagement are now paramount. Individuals work within a matrix of co-worker relationships at work, and kin relationships at home. Each of these relationships constitutes border-keeper expectations, so knowledge workers must craft a careful path between competing expectations. *Megan* paid more attention to work than family. She worked while on vacation (an extreme example in our study), to avoid out-of-control work situations.

Megan: No. I’ll even take meetings while I’m on leave too. And I’ve always told my team, you rather bug me while I’m on holiday and don’t let something escalate out of control, because even though I’m on holiday, I can still manage it. *(Middle manager, full-time, age 40–44, teleworker all week).*

## Discussion

Our article exposes the true nature of the work–life interface for flexible knowledge workers. In the two decades since boundary theory and border theory were first proposed, the rise of ICT has drastically altered the work–life interface. Work can now be completed at any time and in any location, meaning that domains are more likely to be blended and boundaries barely exist (as we have argued above). This implies boundary theory and border theory should now be reappraised in the context of knowledge work. Due to the difficulty in grasping the concept of boundarylessness, work–life researchers have tended to classify work and non-work domains into bounded categories (Allen et al., 2014). While firm boundaries may still apply for certain types of workers and industries, the flexible knowledge workers in our study have revealed that a new form of boundary theory must be formulated. By highlighting the work–life interface and boundary management strategies of knowledge workers, our research contributes to understanding of knowledge work, as well as the objective, subjective, and temporal experiences of knowledge workers who traverse multiple work–life boundaries in a day. Based on our findings, we also argue that the segmentation-integration continuum put forth by Ashforth et al. (2000) boundary theory may be more complicated for knowledge workers than how it is currently conceptualized. We thus call upon work–life researchers to adopt similar qualitative designs grounded in the work–life experiences of knowledge workers in future studies to advance knowledge and theory in this area.

Researchers also need to elaborate the concept of work–home conflict. Though role strain theory predicts conflict, in this study participants were less likely to regard their working lives and home lives as a source of conflict. The introduction of mobile communication technologies and the intersection with flexible working imply that conflict is differently comprehended by flexible knowledge workers. Work devotion may be more salient for individuals, but the level of work devotion does not necessarily imply greater control across the work–life interface. Individuals often prioritize work demands over home demands, suggesting that individuals have less control. This problem is exacerbated for teleworkers, where in theory it is possible for the individual to ignore work demands in favor of home demands. This does not happen: individuals make work a higher priority, most of the time, with limited exceptions for family time or personal time at specific moments of the working day.

### Implications for Human Resource Management

There are four important implications for HR, as flexible knowledge workers navigate the boundaryless work–life interface. First, HR must shape a positive organizational culture that supports flexible work, advocating benefits of FWAs with leaders. HR should challenge organizational practices equating organizational commitment with face-time. Giving knowledge workers flexibility in terms of where and when to work has been shown to alleviate work–family conflict (Golden et al., 2006). Further, HR should instill new practices measuring output, mostly virtual in substance. Productivity must be measured by something different—for example, quality of presentations at virtual meetings.

Second, HR managers must provide meaningful, universal access to FWAs. HR should introduce these policies in organizations that have none, and, in organizations where FWAs are restricted, HR should rewrite policies to include all job families and management levels.

Third, HR must establish procedures making managers, not employees, accountable for tailoring suitable FWAs. Shifting focus to managerial accountability forces managers to be more engaged in making flexible working successful for both employee and supervisor. It would also ensure managers are committed to team success and flex-worker success, rather than leaving employees to muddle through and make it work individually.

Finally, HR must work with information technology (IT) leaders to ensure appropriate balance between technology provisioning, and work and home demands. ICTs are a double-edged sword: on the one hand, empowering employees and enabling work; on the other hand, forcing employees to be constantly connected and constantly available. HR and IT should define appropriate expectations about technology usage by employees. Managers should avoid unwittingly setting an expectation of 24/7 availability, by for example, answering emails late at night. HR should provide pragmatic guidelines: if employees have to collaborate across time zones, it is necessary to work early or late, but employees should feel they have time to disconnect and recover. The appropriate flexible work policy framework, situated in a flex-positive organizational culture, with leader, HR and IT role modeling and resourcing, sets the organization up for success.

### Limitations and Directions for Future Research

There are several limitations to this study. The cross-sectional research method did not allow investigation of the construction of the work–life interface over time. From a sampling perspective, the study did not deliberately include full-time co-workers. Given the nature of flexible work, full-time employees were the largest group within Tech, so their perceptions and behaviors influence organizational perceptions of those who work flexibly. The study did not account for the influence of cultural background such as national culture or ethnic background. Furthermore, the study did not account for generational influences on flexible working perceptions. It is reasonable to assume younger employees may have different expectations about work–life balance and how they might construct the work–life interface. Positive response bias is a particular concern when using semi-structured interviews. Though steps were taken to avoid this kind of bias, further triangulation of the findings could help to improve authenticity.

There are interesting future directions for research involving flexible knowledge workers and their work–life interface. First, researchers should compare and contrast full-time workers with flexible workers who vary hours, schedule or location of work in future studies. Second, seeing that manager support was essential to positive flexible working perceptions, it would be worthwhile to investigate the specific role of managers and supervisors. Some sample questions include: How do effective managers support subordinates and what do less effective managers do or not do?

Another direction for future research is to look more closely at the host location of the supervisor compared to the subordinate. If supervisor and subordinate are in the same office location, then there is more opportunity for interaction, but it also carries an expectation that the employee will be present in the office, unless they have an agreement with the manager for remote or flexible working. If the supervisor is in a different office location, then face-to-face supervision is not possible and expectations might be different. Furthermore, the employee might be forced to work outside conventional hours in order to have discussions with the direct supervisor who may be on the other side of the world.

Conceptual definitions of flexible working are also problematic. The precise definition of flexibility, and the intensity and duration of flexible working are important factors in studying the work–life interface. Conceptual definitions are not consistent in the literature so future studies could work toward a consistent and widely accepted definition. Also, given that teleworking appears to have fewer negative consequences compared to working reduced (part-time) hours, future studies should look at the mechanisms that drive these varying outcomes across different cohorts and types of FWAs.

Finally, future research should investigate other industries and other countries, to expand research and scholarship about flexible working into new sectors. It is simple for an IT firm to offer flexible working to knowledge workers, because knowledge work is portable—other types of industries such as retail, manufacturing and healthcare would have to offer FWAs to selective occupational groups. Comparative research between industries presents novel opportunities for theory building and analysis.

## Conclusion

In the last decade, work and life have been transformed by technology, fragmenting time. Now, knowledge workers can work anywhere, at any time. This brings unprecedented empowerment—yet, simultaneously, enslavement. Existing boundaries are no longer salient for flexible knowledge workers. They perceive their work–life interface as fuzzy and boundaryless. Knowledge workers use ICTs combined with FWAs to craft their ideal lives. In bringing attention to fuzzy boundarylessness, we hope to guide HR practitioners, and leaders, to develop new HR approaches for flexible knowledge workers. We encourage future studies to examine the fuzzy boundaryless nature of the work–life interface and explore the different ways flexible knowledge workers navigate work and home, family and life.

## Ethics Statement

This study was carried out in accordance with the recommendations of the Human Research Ethics Committee of the University of New England, with written informed consent of all subjects.

## Author Contributions

JF conceptualized the boundaryless work–life interface and collected the qualitative data. XC analyzed the literature and came out with the overall structure. Both authors made a substantial, direct and intellectual contribution to the work.

## Conflict of Interest Statement

The authors declare that the research was conducted in the absence of any commercial or financial relationships that could be construed as a potential conflict of interest.

## Appendix 1: Interview Template

Questions regarding absences, flexible working and ICT:

1. What type of absence did you take? (maternity, paternity, leave of absence, other types)
2. OR, What type of flexible working arrangement did you seek? (part-time in the office, work from home part-time or full-time)
3. What event or scenario triggered your request for absence or flexible working?
4. When and how did you involve your manager? What happened?
5. What information and communication technologies do you use? Why? Which ones do you avoid and why?
6. If demographic information indicates that the employee has caring responsibilities for family, ask: at home, what household or caring responsibilities do you have? How do you balance work versus home and family?
7. When you were absent, did you feel you needed to try to keep in touch and how did you do this? What happened?
8. What happened when you returned to work after absence? What did your manager say and do?
9. If you’re working part-time and/or working at home all of the time or some of the time, how do you define what is work time and what is non-work time? Have there been any times when you’ve felt under pressure from one side or the other? What happened? What does your manager say and do? What do your family and friends say and do?

For participants who indicated they were working from home:

1. Do you ever work very early in the morning or very late at night? Why? Is this your choice or has your manager directed you?
2. Where are you more productive, at home or in the office?
3. Where do you receive more interruptions?
4. Where do you feel more distracted?
5. What health concerns do you have about working from home?
6. To what extent do you feel lonely working from home?
7. To what extent do you feel isolated working from home?
8. To what extent do you feel you are missing out on office gossip, the grapevine or other informal communications? How important is this to you?
9. How do you blend work tasks with housework and childcare duties, when working from home?
10. For participants with dependent children: who does pickup and drop-off?
11. What do your family and friends say when you mention you work from home?
12. When do you make yourself strictly unavailable?
13. Do you ever disconnect or turn off your laptop or mobile phone?

For participants who indicated they were working part-time:

1. What days of the week do you work and how did you choose those days? How has this arrangement changed over time?
2. To what extent do you have time to socialize on a working day?
3. To what extent do you feel you are missing out on office gossip, the grapevine or other informal communications? How important is this to you?
4. To what extent do you work on a non-working day? Why? Is this your choice or has your manager directed you to work on a non-working day?
5. What is the value, to you, of your non-working day?
6. What pressure have you had to change to full-time employment?
7. How many other part-time employees do you know?
8. For participants with dependent children: who does pickup and drop-off?
9. When do you make yourself strictly unavailable?
10. Do you ever disconnect or turn off your mobile phone?

For participants who mentioned an unpaid leave of absence, usually in the form of a sabbatical:

1. What was the response of co-workers when you announced the leave of absence?
2. What happened during the time away from work?
3. How important was it to keep in touch with work while you were away? What did you do to keep in touch?
4. What benefits did the leave of absence give you?
5. What negative consequences did the leave absence give you?
6. How did you arrange to come back to work?
7. What was the response of co-workers when you returned to work?
